# Network pharmacology‑based investigation of potential targets of triptonodiol acting on non-small-cell lung cancer

**DOI:** 10.1186/s40001-023-01453-4

**Published:** 2023-11-28

**Authors:** Feng Jin, Xiaochen Ni, Shilong Yu, Xiaomin Jiang, Jun Zhou, Defang Mao, Yanqing Liu, Feng Wu

**Affiliations:** 1 Department of Respiratory and Critical Care Medicine, The Affiliated Hospital of Yangzhou University, Yangzhou University, Yangzhou, Jiangsu People’s Republic of China; 2 Institute of Translational Medicine, Medical College, Yangzhou University, Yangzhou, 225001 People’s Republic of China; 3 The Key Laboratory of Syndrome Differentiation and Treatment of Gastric Cancer of the State Administration of Traditional Chinese Medicine, Yangzhou, 225001 People’s Republic of China; 4 Yangzhou Hospital of Traditional Chinese Medicine, Yangzhou, 225001 People’s Republic of China

**Keywords:** Natural products, Network pharmacology, *Tripterygium wilfordii*, Phytochemicals

## Abstract

**Background:**

Triptonodiol is a very promising antitumor drug candidate extracted from the Chinese herbal remedy *Tripterygium wilfordii* Hook. F., and related studies are underway.

**Methods:**

To explore the mechanism of triptonodiol for lung cancer treatment, we used network pharmacology, molecular docking, and ultimately protein validation. Gene ontology (GO) analysis and Kyoto Encyclopedia of Gene and Genome (KEGG) pathway enrichment analysis were performed through the David database. Molecular docking was performed using PyMoL2.3.0 and AutoDock Vina software. After screening, the major targets of triptonodiol were identified for the treatment of lung cancer. Target networks were established, Protein–protein interaction (PPI) network topology was analyzed, then KEGG pathway enrichment analysis was performed. Useful proteins were screened by survival analysis, and Western blot analysis was performed.

**Results:**

Triptonodiol may regulate cell proliferation, drug resistance, metastasis, anti-apoptosis, etc., by acting on glycogen synthase kinase 3 beta (GSK3B), protein kinase C (PKC), p21-activated kinase (PAK), and other processes. KEGG pathway enrichment analysis showed that these targets were associated with tumor, erythroblastic oncogene B (ErbB) signaling, protein phosphorylation, kinase activity, etc. Molecular docking showed that the target protein GSK has good binding activity to the main active component of triptonodiol. The protein abundance of GSK3B was significantly downregulated in non-small-cell lung cancer cells H1299 and A549 treated with triptonodiol for 24 h.

**Conclusion:**

The cellular-level studies combined with network pharmacology and molecular docking approaches provide new ideas for the development and therapeutic application of triptonodiol, and identify it as a potential GSK inhibitor.

## Introduction

With the change in living habits and the increase in global emissions, lung cancer has become the most common cancer in terms of incidence and mortality worldwide. 85 percent of lung cancers in the histologic classification are non-small-cell lung cancers and share similar biological features. Due to the lack of early diagnostic techniques and the nonspecific or even asymptomatic nature of early clinical symptoms in lung cancer patients, lung cancer patients often have progressed by the time they are diagnosed [1]. Chemotherapy is the first-line treatment option for lung cancer and has been successfully and widely used in the clinic. However, the therapeutic efficacy of chemotherapy is limited by the lack of specific biomarkers for identifying those patients who may benefit from chemotherapy [2]. Nowadays, about 1.6 million people die of lung cancer every year, so the search for new low-toxicity and effective anti-lung cancer drugs is a matter of great urgency [3].

Traditional Chinese medicine has a long history of application in China and plays an important role in treating tumors [4]. After billions of years of screening by nature, natural compounds have richer structures and higher biological activities and are gradually becoming an important resource library for drug development [5]. Herbal medicines (including extracts and compounds) exert antitumor effects through multiple pathways and multiple targets, but in most cases, their targets cannot be fully elucidated [6]. Revealing the direct targets of these natural compounds has the opportunity to discover new drug targets while searching for promising drugs and expanding the library of lead compounds. *Tripterygium wilfordii* is a Chinese herbal medicine that is often used to treat tumors in China. Early research by our team identified one of the compounds, triptonodiol, as having excellent antitumor efficacy [7]. However, its molecular mechanism remains unknown. Therefore, this study used network pharmacology combined with molecular docking to predict whether triptonodiol could act on lung cancer targets. Western blot experiments were performed to validate the true pharmacological activity of the predicted target, and the prognostic value of the target in the clinic was analyzed using TCGA data. This study provides the first systematic study of the action and mechanism of triptonodiol, and provides an experimental basis for the application of triptonodiol as an antitumor drug candidate.

## Methods

### Collection of triptonodiol and lung cancer-related genes

The molecule structure of triptonodiol was portrayed using PubChem [8] database (https://pubchem.ncbi.nlm.nih.gov/) and the SDF format was preserved. The structural information of triptonodiol was imported into Swiss Target Prediction database (http://www.swisstargetprediction.ch/) [9] and PharmMapper database (http://www.lilab-ecust.cn/). pharmmapper/) to obtain potential targets for triptonodiol. Lung adenocarcinoma (GSE118370) and squamous lung cancer (GSE3268) datasets were searched from the GEO database (https://www.ncbi.nlm.nih.gov/geo/), gene expression differences between tumor tissues and paracancerous tissues were analyzed, and differential genes were merged and constructed into a database of non-small-cell lung cancer disease targets.

### Prediction of triptonodiol’s anti-lung cancer targets

Using the Venny 2.1 (https://bioinfogp.cnb.csic.es/tools/venny/) online too, the potential targets of triptonodiol were compared with the relevant genes of lung cancer. Venn diagrams were drawn, and the intersection of the two was the potential therapeutic target of triptonodiol against lung cancer.

### Construction of protein–protein interaction network and its analysis

The crossing point of potential triptonodiol targets and lung cancer-related qualities was imported into STRING database [10] (https://string-db.org, Version 11.0), ‘‘Organism’’ was set as ‘‘Homo sapiens’’, set the ‘‘minimum required interaction score’’ to 0.400, avoided the free target qualities, developed the PPI network [11], traded the tsv record of the arrange, and imported the tsv record into Cytoscape (Version 3.9.0) software [12] was utilized to imagine and analyze the PPI network.

### GO enrichment analysis and KEGG enrichment analysis

The important genes of lung cancer and potential helpful targets of triptonodiol were imported into the DAVID [12, 13] database (https://david.ncifcrf.gov/, Version 8.0) [14], ‘‘Identifier’’ was chosen as ‘‘ GENE_OFFICIAL_SYMBOL’’, ‘‘species’’ was characterized as ‘‘Homo sapien’’, and GO enrichment investigation and KEGG enrichment examination were performed. Both GO and KEGG improvement investigation were performed with the screening condition of *P* < 0.05 [15], and the three aspects of biological process (BP), cellular ingredients (CC) and molecular function (MF) of the significant targets were examined, and the bar graphs of enrichment analysis were drawn.

### Target analysis of triptonodiol for lung cancer

The KEGG PATHWAY Database (https://www.genome.jp/kegg/pathway.html) was used to search for signaling pathways and annotate the genes in the pathways. The predicted triptonodiol anti-lung cancer targets were compared with the pathway-related genes, and the intersection was taken to identify the triptonodiol anti-lung cancer enzymatic targets.

### Molecular docking

We performed atomic docking examination. 2D structures of the dynamic compound were downloaded from the PubChem database, and their 2D structures were optimized utilizing Chem3D. The 3D maps of the core proteins were retrieved and downloaded from the PDB database. The target proteins were dewatered, and the original ligands were removed by PyMOL software. Molecular docking was performed using Autodock visualization software [16]. The target proteins were dewatered, and the first ligands were evacuated by PyMOL program. Atomic docking was performed utilizing Autodock visualization program.

### Analysis of gene prognosis of drug targets

Survival investigation was performed on the EGPIA2 site (http://gepia2.cancer-pku.cn/#index) by entering the target genes of interest and selecting tissue samples for lung adenocarcinoma (LUAD) and lung squamous carcinoma (LUSC). 50% of Cutoff-High and 50% of Cutoff-Low each. The TCGA database contains expression and clinical information on 33 sorts of tumors.

### Cell culture

H1299 cells and A549 cells were purchased from Procell Life Science&Technology Co., Ltd, and cell culture was performed in strict accordance with the manual provided by the company. H1299 cells were maintained in culture using RPMI-1640 (Cytiva, Catalogue Number: SH30809.01) and A549 cells were maintained in culture using Ham's F-12K (Procell, Catalogue Number: PM150910). The culture medium was supplemented with 10% fetal bovine serum (Gibco, Catalogue Number: 10099-141) without any antibiotics. The incubator parameters were set at 37 ℃, 5% CO2, and saturated humidity.

### Preparation of drugs

Triptonodiol was purchased from MedChemExpress (MCE, Catalogue number: HY-N1121; CAS No.: 117456-87-8) and dissolved in DMSO. The highly concentrated solution (100 mM) was stored in a − 20 °C refrigerator in a brown, lightproof vial. When required for use, dilute with culture medium to the desired concentration. The concentration of DMSO in the medium is always less than 0.1%.

### Western blot

After COE treatment of NSCLC cells for 24 h, the cells were collected and lysed with RIPA buffer, and total cellular protein was extracted, followed by quantification using the BCA kit. Cell lysates were mixed with loading buffer and subjected to vertical gel electrophoresis on SDS-PAGE, followed by horizontal electrophoresis to transfer the proteins to PVDF (Millipore) membranes. PVDF membranes were blocked with buffer containing 10 mmol/L Tris, 150 mmol/L NaCl, 0.1% Tween-20 and 5% skimmed milk, then incubated with primary antibody(Cell Signaling Technology, GSK3B, 12456S, 1:1000 dilution) at 4 °C overnight (usually 14 h), followed by incubation with a cognate secondary antibody coupled with horseradish peroxidase for 1 h at room temperature. Internal reference was controlled by GAPDH. Immunoreactive bands were detected using an enhanced chemiluminescence gel imaging system (Bio-Rad).

### Statistical analysis

Statistical analysis was performed using SPSS and differences between three and more groups were analyzed using one-way ANOVA. The differences were considered to be statistically significant when *P < 0.05, **P < 0.01, and ***P < 0.001.

## Results

### Screening of the potential targets of triptonodiol in treating lung cancer

**Fig. 1:**
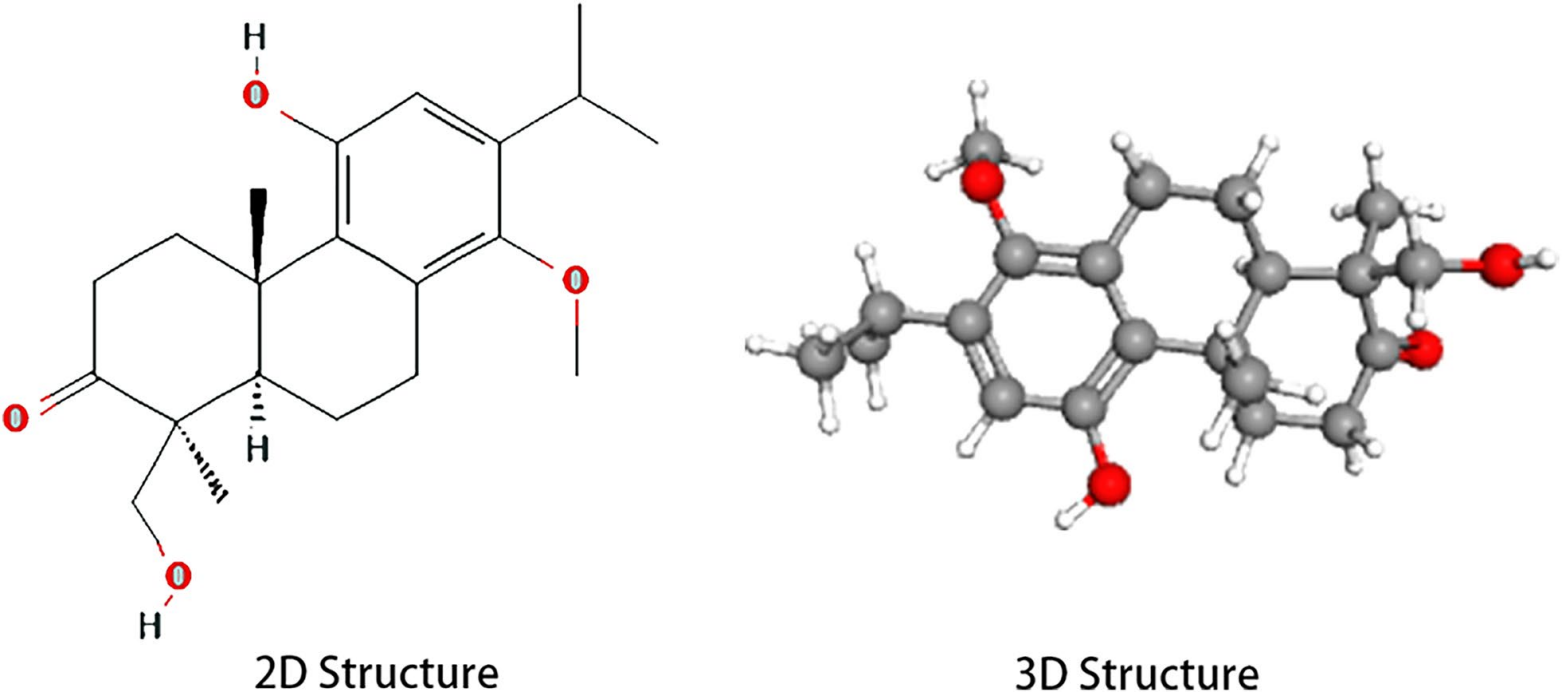
The 2D and 3D structures of triptonodiol

**Fig. 2:**
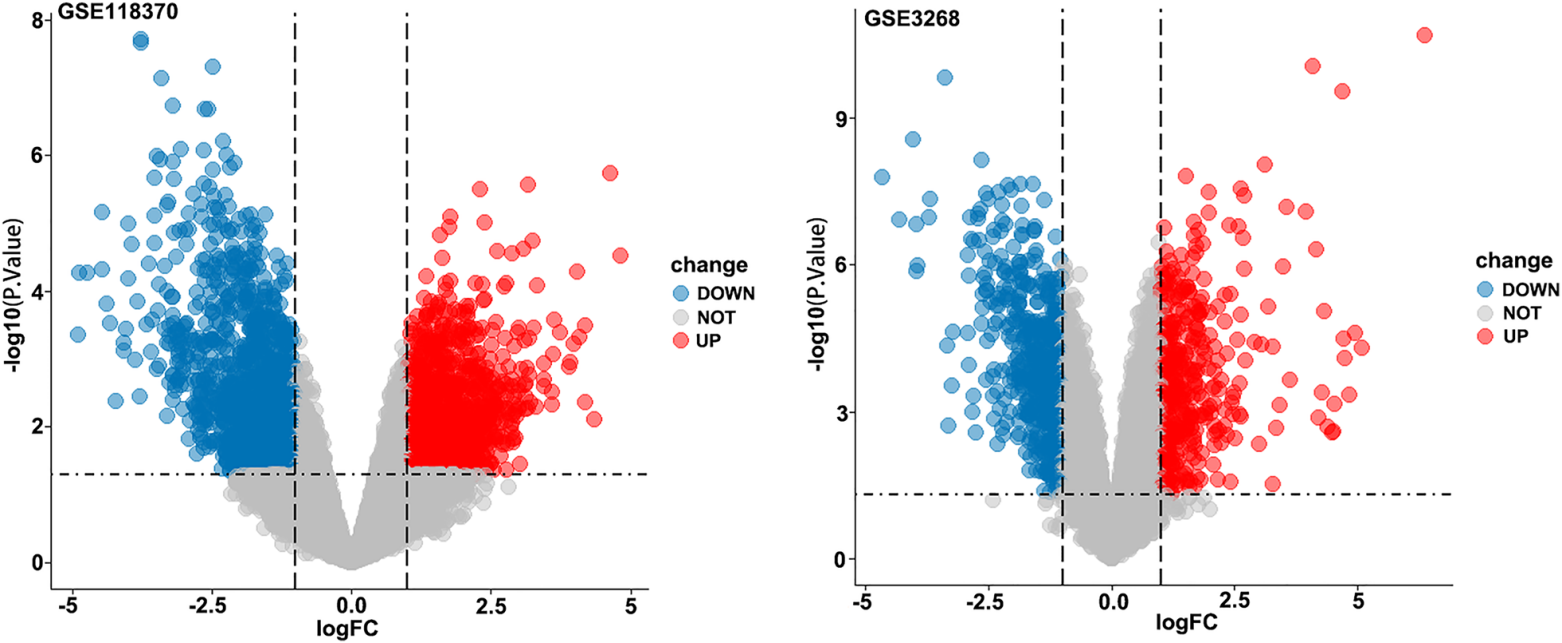
Volcano plot of differentially expressed genes in lung cancer. The horizontal coordinates indicate the fold relationship of gene expression changes and the vertical coordinates indicate the statistical significance of gene expression changes. Red dots represent genes with significantly upregulated expression and blue dots represent genes with significantly downregulated expression

**Fig. 3:**
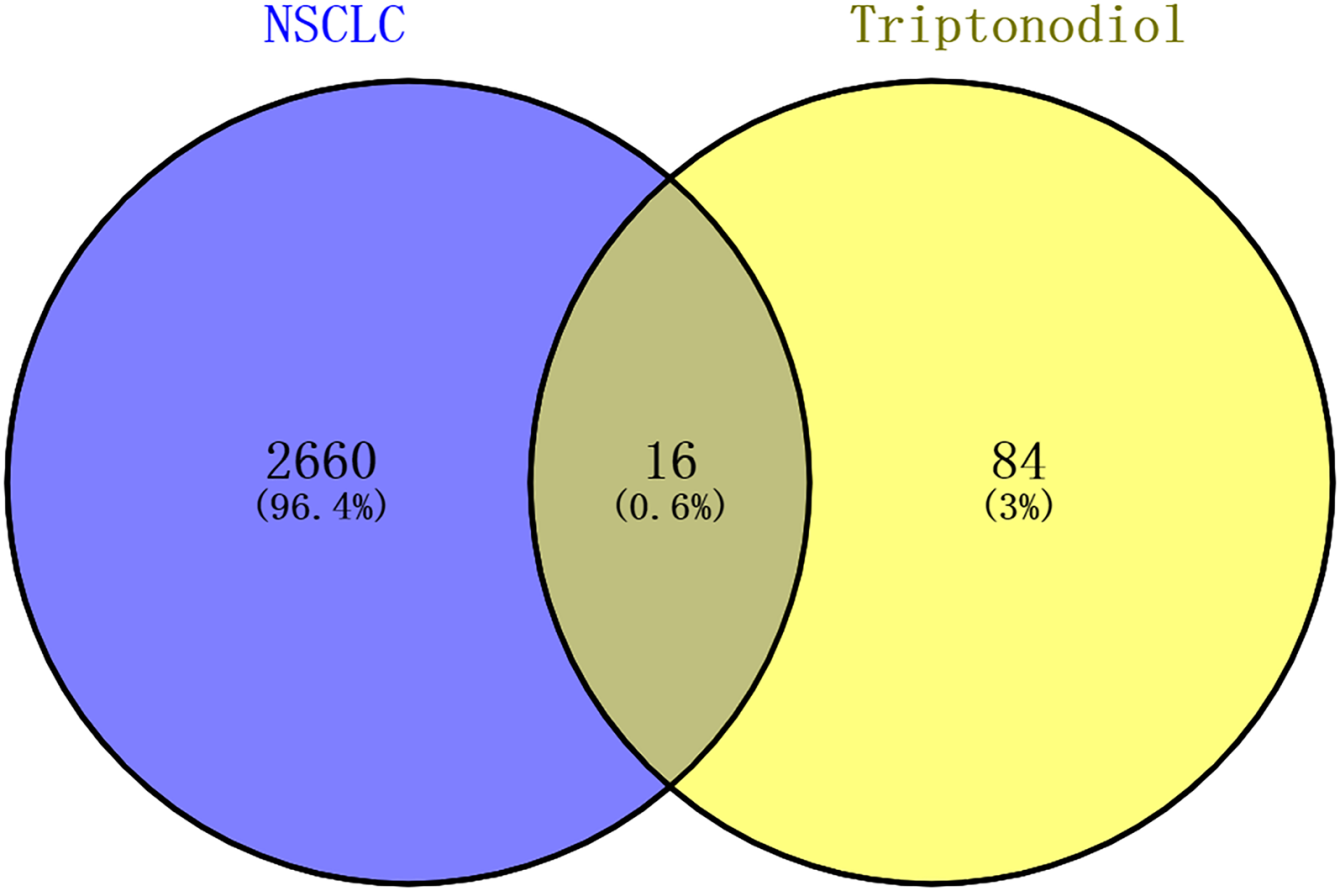
Venn diagram of the targets of the active ingredients of triptonodiol and the lung cancer-related targets

The 2D structure of triptonodiol was acquired from the Pubchem database (Fig. 1) and entered into the SwissTargetPrediction website, which yielded a total of 100 potential targets. Genes related with lung cancer were mapped as volcanoes, which were identified from the GEO database. A total of 1158 genes were upregulated and 1212 genes were downregulated in lung adenocarcinoma (GSE118370). A total of 386 genes were upregulated and 507 genes were downregulated in squamous lung cancer (GSE3268) (Fig. 2). After combining and deleting duplicates, 2676 genes were obtained as disease targets in non-small-cell lung cancer. Based on the screened components and their targets, the intersection of drug and disease targets (Fig. 3) was taken, and a total of 16 genes were obtained, which will be used as potential targets of triptonodiol for lung cancer treatment in subsequent network construction and pathway enrichment analysis.

### The core target of triptonodiol in the treatment of lung cancer

**Fig. 4:**
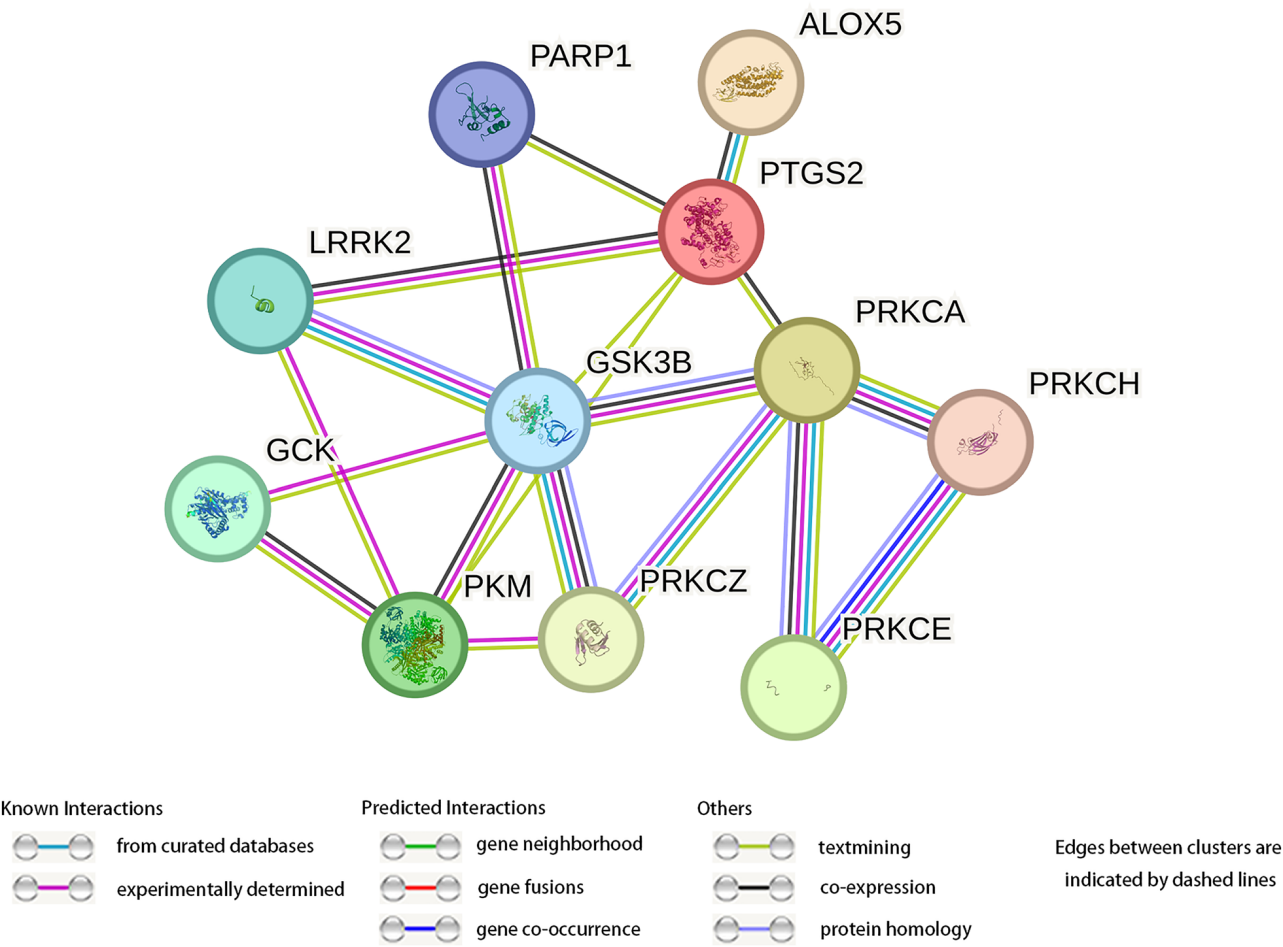
PPI network of the common targets of triptonodiol and lung cancer

**Fig. 5:**
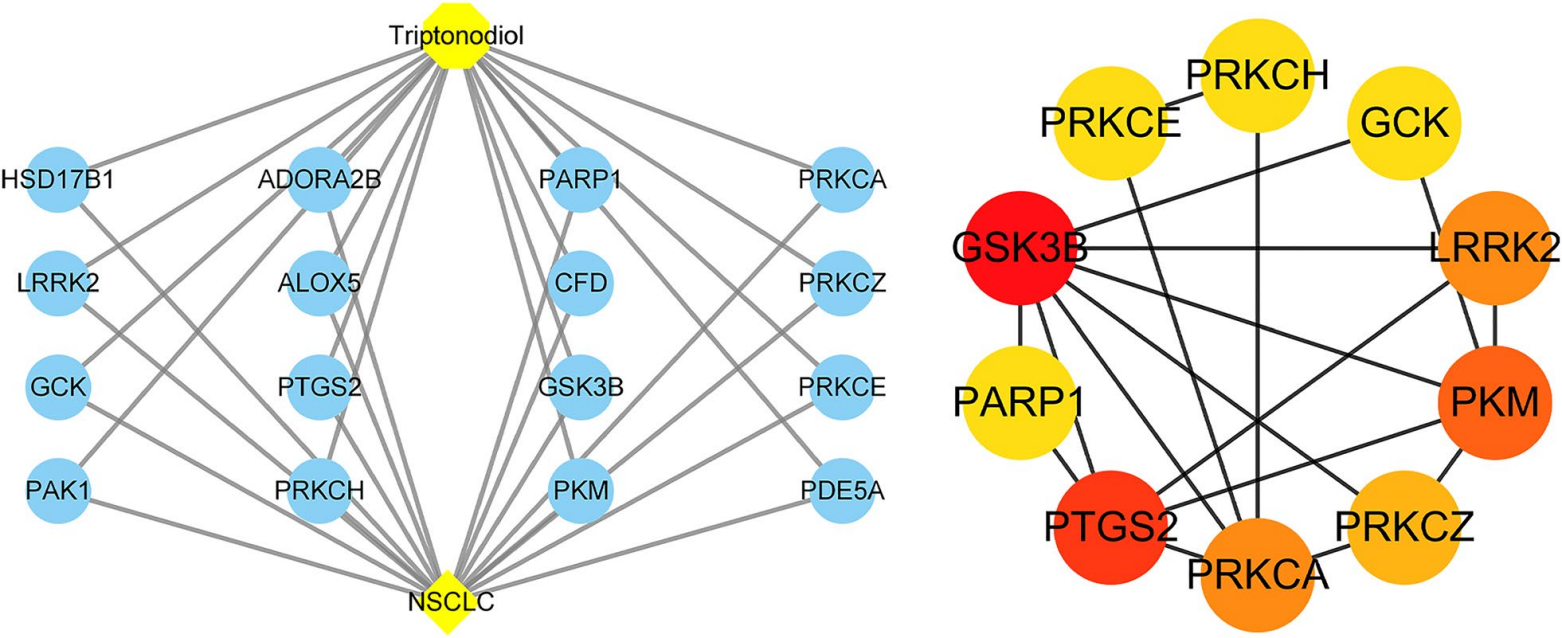
Ingredient-target network of triptonodiol and the network of the first 10 genes

The potential targets of triptonodiol for lung cancer were entered into the STRING database with 16 nodes to obtain the PPI network (Fig. 4). The node with Degree > 2 times the average number of neighbors is regarded as the key node in the network, and then 10 key proteins were screened out (Fig. 5). Among them, GSK3B is the highest-scoring one, with degree values of 14. Among the top ten scoring genes, GSK3B, PRKCA, PRKCZ, PRKCH, and PRKCE play a role in the ErbB signaling. Also, the gene PAK1, scored at 12th, is an important regulator in the ErbB signaling. These results suggest that triptonodiol is likely to play a role by regulating the ErbB signaling pathway and has potential in the treatment of lung cancer.

### GO and KEGG enrichment analysis

**Fig. 6:**
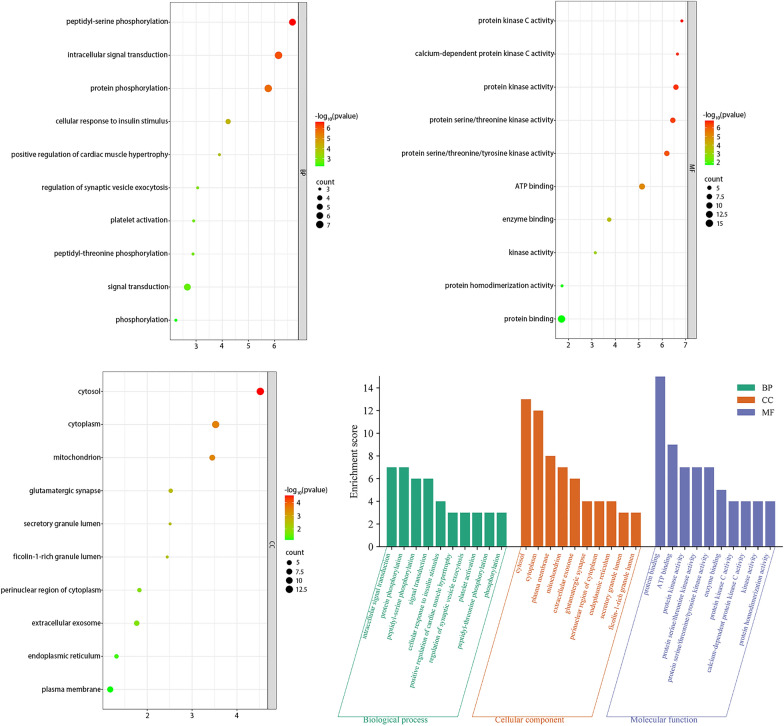
Gene Ontology terms of candidate targets of triptonodiol against lung cancer. The top 10 GO functional categories with FDR < 0.05 were selected

**Fig. 7:**
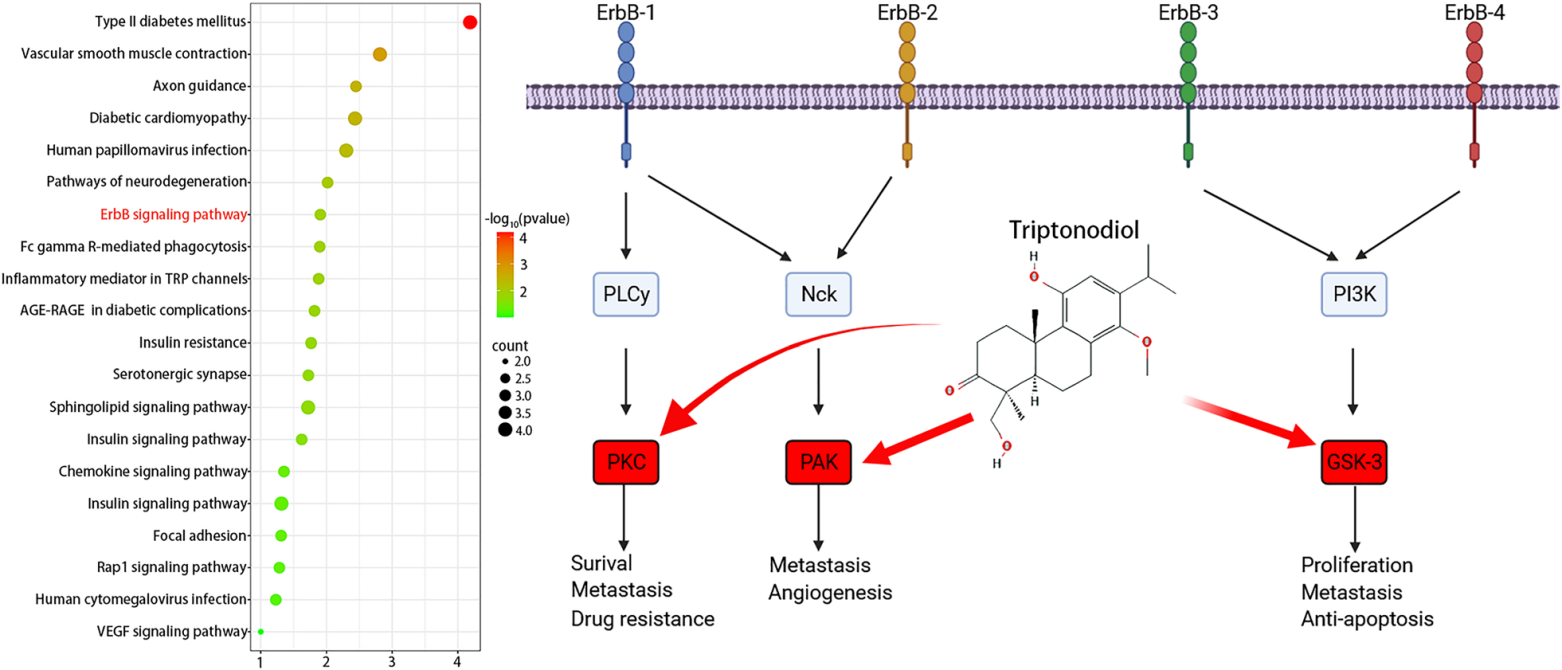
KEGG pathway enrichment of candidate targets of triptonodiol against lung cancer. Pathways that had significant changes of FDR < 0.05 were identified. The size of the spot represents a number of genes and color represents FDR value. The most enriched pathways are shown in the ErbB signaling diagram on the right, with the top ten gene targets for screening marked by red boxes

GO and KEGG pathway enrichment analysis of the identified core targets of triptonodiol for lung cancer by DAVID software. Cellular composition and molecular functional analysis of the candidate targets were performed based on biological processes. As shown in Fig. 6, GO terms that are exceedingly enriched in biological processes, cellular components, and molecular functions incorporate cellular signaling, protein phosphorylation, and protein kinase activity. Then, the target genes were analyzed by KEGG enrichment investigation, and the 20 signaling pathways with the highest enhancement scores were positioned (Fig. 7). We found that the highest scoring tumor-associated signaling was the ErbB signaling pathway, and triptonodiol may act by directly targeting PKC, PAK, and GSK3B, which is consistent with the results of the PPI network. These results suggest that triptonodiol may be a promising antitumor agent.

### Analysis of gene prognosis of drug targets and molecular docking

**Fig. 8:**
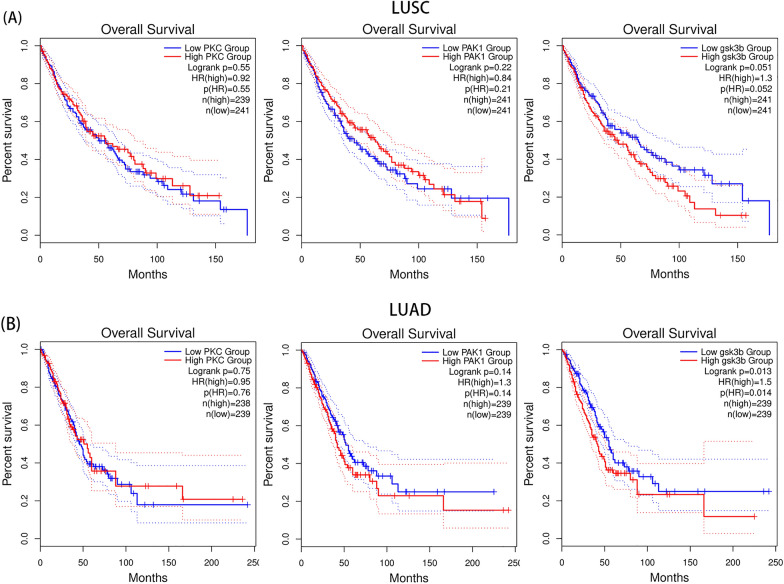
Prognostic curves of four hub genes. The prognostic significance of the hub genes in patients with lung cancer, according to the TCGA database. A: PKC, PAK1, GSK3B in squamous lung cancer. B: PKC, PAK1, GSK3B in lung adenocarcinoma

**Fig. 9:**
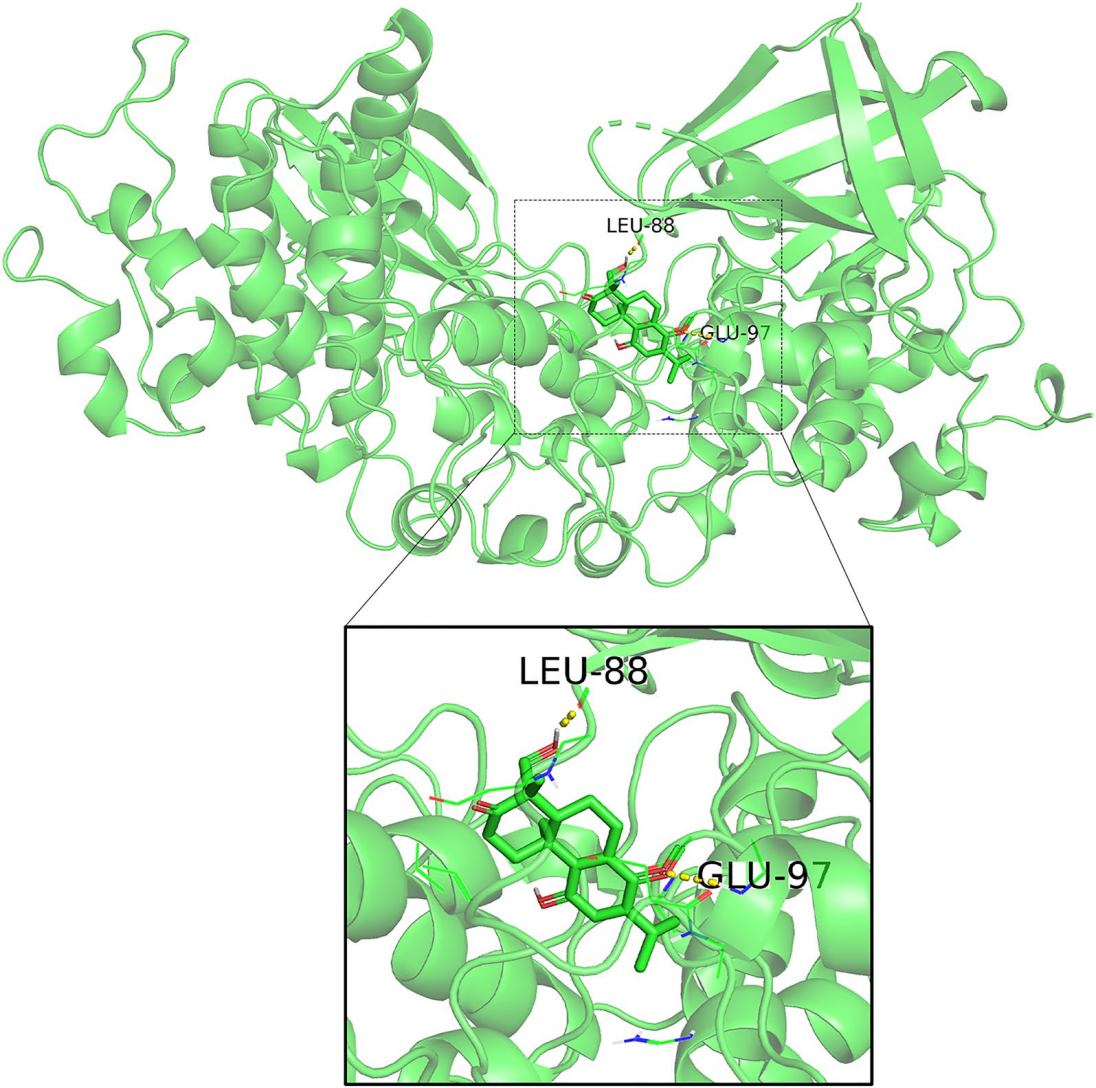
Molecular docking patterns

Genetic prognostic analysis of drug targets was performed using the TCGA tumor database, which contains expression and clinical data for 33 tumors. We analyzed the prognostic impact of PKC, PAK1, and GSK3B in patients with lung adenocarcinoma and squamous lung cancer. Among these targets, GSK3B had a significant effect on lung adenocarcinoma patients, and the statistical analysis of the effect on squamous lung cancer patients was not very significant (Fig. 8). Therefore, the molecular docking simulations of triptonodiol and GSK3B were performed. An absolute value of free binding energy greater than 4.2 prompts binding activity, greater than 5.0 prompts good binding activity, and greater than 7.0 prompts strong binding activity [17]. The absolute value of free binding energy for triptonodiol and GSK3B was 6.35, indicating good binding activity (Fig. 9).

### Western blot

**Fig. 10:**
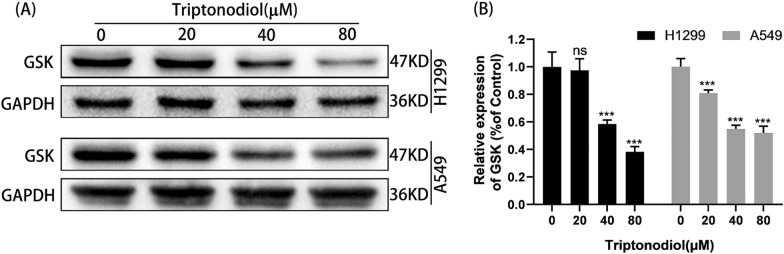
Western blot. After treatment of triptonodiol for 24 h, the protein levels of GSK were significantly downregulated

Network pharmacology can significantly improve the hit rate of drug target screening, but it is still a virtual screening tool. To finally confirm the biological activity of triptonodiol against GSK3B, the protein level of GSK3B was detected by Western blot. Excitingly, the protein levels of GSK3B were significantly downregulated after 24 h of treatment of non-small-cell lung cancer H1299 and A549 with the indicated concentrations of triptonodiol (Fig. 10). GSK3B is an effector downstream of ErbB signaling that plays an important role in tumor proliferation by directly regulating P21 and Cyclin D1. The above results suggest that triptonodiol is a highly promising antitumor agent that inhibits the biological activity of GSK3B.

## Discussion

Currently, tumors are one of the most serious diseases endangering human wellbeing. It is evaluated that by 2019, the number of tumor-related deaths will surpass 20 million around the world. The frequency of tumors in China is additionally on the rise year by year [18]. Chinese traditional medicine plays an imperative role in the prevention and treatment of tumors, and its role and mechanism in enhancing the immune function of the body, preventing the multidrug resistance of tumor cells, restraining the division and expansion of tumor cells, and accelerating the apoptosis of tumor cells are gradually revealed. The foundation for its effective application in antitumor therapy has been laid [19]. Be that as it may, the composition of Chinese traditional medications is complex, and their active ingredients are still unclear in pharmacology and clinical practice. The isolation of the monomers among them is time-consuming and costly, and the specific target genes or proteins for drug action have not been fully identified. In the past decade, with the fast improvement of high-throughput innovations and bioinformatics, the research of network pharmacology in disease treatment has attracted attention. Based on computer simulation technology, it provides an effective method for component screening and prediction of drug targets, which is a feasible solution to improve the correct rate of drug target prediction [20].

*Tripterygium wilfordii* has been used in China for at least several hundred years to treat a variety of tumor diseases, and the earliest detailed records can be found in the Qing Dynasty book “Supplement to Compendium of Materia Medica”. *Tripterygium wilfordii* is a renewable resource with huge reserves and has been found growing in 1219 regions worldwide [21]. The use of traditional Chinese medicine (TCM) for the treatment or adjuvant therapy of tumors in China has developed into a distinctive Chinese therapeutic strategy and has been shown to have significant therapeutic efficacy [22–24]. Triptonodiol is one of the compounds in *Tripterygium wilfordii*, and our preliminary studies have demonstrated its antitumor effects [7]. In this study, we further examined the mechanism of activity of triptonodiol in lung cancer, and 16 key targets were identified. In addition, we used PPI to explore data mining and network analysis. We found 10 core targets, which are exceedingly connected with tumor resistance and proliferation.

GSK-3 is display in all eukaryotes and could be a broadly expressed and exceedingly conserved serine/threonine protein kinase that can be inactivated by p70S6k through serine 9/21 phosphorylation [25–27]. EMT can be positively regulated by the Wnt/β-catenin pathway [28], and GSK-3 plays an imperative administrative role in this process. Active GSK-3β can prevent transcription of β-catenin target genes by stimulating the degradation of β-catenin protein and promoting the destruction of nuclear phosphorylated β-catenin [29, 30]. At the same time, we carried out KEGG enrichment analysis on the predicted target. The calculated results of KEGG enrichment are in high agreement with those of previous analyses. Triptonodiol is highly correlated with tumor signaling, especially ErbB signaling. ErbB receptors were linked to human cancer pathogenesis approximately thirty years ago. The transmembrane receptor tyrosine kinases (RTKs) of the ErbB family include ErbB1, ErbB3, ErbB2, human epidermal growth factor receptor-2 (HER2), and HER4 [30]. ErbB family members play an important role in the occurrence and maintenance of various solid tumors, especially HER2-amplified breast cancer and epidermal growth factor receptor (EGFR) mutated lung cancer. This led to the development and widespread implementation of specific ErbB inhibitors as cancer therapies. At the same time, particularly in lung cancer, mutations in ErbB4 have been identified [32]. The mutation in the gatekeeper residue of EGFR, T790M, is the most common mechanism of acquired resistance to EGFR inhibitors in EGFR mutant lung cancer. At least 50% of biopsies from patients with acquired drug resistance carry the T790M mutation of EGFR [33, 34]. It is reported that the S492R mutation occurred in the EGFR extracellular domain, which can interfere with the binding of cetuximab [35]. In addition to the immune effect of the ErbB antibody, most of the activities of many similar drugs are due to the inhibition of downstream signal transduction, especially PI3K/AKT/GSK-3 and MEK/ERK [31]. Therefore, although the targeted receptor is completely inhibited, at least one of these key downstream pathways is still maintained, so many cancers are resistant to ErbB inhibitors. This type of drug resistance, also known as "bypass track" resistance, is usually used to describe the drug resistance caused by maintaining these key downstream signaling when RTK is fully inhibited [36]. In addition, mutations in the target are also important factors in acquired resistance, such as the most common EGFR mutation [37]. Combination of vascular endothelial growth factor (VEGF) inhibitors is an advantageous strategy in non-small-cell lung cancer patients with EGFR mutations [38]. In our KEGG screen results, triptonodiol may also be an inhibitor of the VEGF signaling pathway. Therefore, the calculation results of triptonodiol by the network pharmacology model have excellent application prospect. Our results show that triptonodiol can regulate ErbB signaling and VEGF signaling at the same time. In theory, it could treat cancer while reducing the incidence of drug resistance, which gives us some room for reverie.

In conclusion, we provided a preliminary exploration of the dominant targets and effective pathways of triptonodiol with the help of network pharmacology and laboratory validation of the protein molecules, confirming the antitumor pharmacological effects of triptonodiol. With the help of network pharmacology, we confirmed that ErbB signaling is the core pathway of triptonodiol's antitumor activity, which could be the basis for the development of new drugs, but more trials are still needed. Since Western blot experiments established that triptonodiol can inhibit the biological activity of GSK in vitro, we next plan to carry out animal pharmacology experiments to observe the effect and mechanism of triptonodiol for NSCLC treatment in depth and provide more references for its clinical application and development.

## Data Availability

All experiments were carried out in the Key Laboratory of Syndrome Differentiation and Treatment of Gastric Cancer of the State Administration of Traditional Chinese Medicine. None of the data comes from third parties.
